# Experience with adjuvant chemotherapy for pseudomyxoma peritonei secondary to mucinous adenocarcinoma of the appendix with oxaliplatin/fluorouracil/leucovorin (FOLFOX4)

**DOI:** 10.1186/1477-7819-6-118

**Published:** 2008-11-11

**Authors:** Chin-Fan Chen, Che-Jen Huang, Wan-Yi Kang, Jan-Sing Hsieh

**Affiliations:** 1 Department of Surgery, Kaohsiung Medical University Hospital, Kaohsiung 807, Taiwan; 2 Department of Pathology, Kaohsiung Medical University Hospital, Kaohsiung 807, Taiwan; 3 Faculty of Medicine, College of Medicine, Kaohsiung Medical University, Kaohsiung 807, Taiwan; 4 Department of Surgery, Pingtung Hospital, Department of Health, Executive Yuan, Ping-Tung 900, Taiwan

## Abstract

**Background:**

Pseudomyxoma peritonei (PMP) is a rare condition characterized by mucinous tumors, disseminated intra-peritoneal implants, and mucinous ascites. So far its diagnosis remains challenging to most clinicians.

**Case presentation:**

A 55-year-old male patient had suffered from acute onset of abdominal pain and abdominal distension for one day prior to his admission. Physical examination revealed tenderness over the right lower quadrant of the abdomen without diffuse muscle guarding. A large amount of ascites was identified by abdominal computed tomography (CT) scan. Paracentesis showed the appearance of sticky mucinous ascites. He underwent laparotomy under the impression of pseudomyxoma peritonei. There was a lot of mucinous ascites, one appendiceal tumor and multiple peritoneal implants disseminated from the subphrenic space to the recto-vesicle pouch. Pseudomyxoma Peritonei caused by mucinous adenocarcinoma of appendiceal origin, was confirmed by histopathology. We performed an excision of the appendiceal tumor combined with copious irrigation and debridement. After the operation, he received 10 cycles of systemic chemotherapy with FOLFOX4 regimen, without specific morbidity. Follow-up of abdominal CT and colonoscopy at post-operative 17 months showed excellent response without evidence of local recurrence or distal metastasis. He made an uneventful recovery (up to the present) for 21 months after the operation.

**Conclusion:**

This case report emphasizes the possible new role of systemic chemotherapy in the treatment of patients with this rare clinical syndrome.

## Background

Pseudomyxoma peritonei (PMP) is a rare condition characterized by mucinous tumors, disseminated intra-peritoneal implants, and mucinous ascites. It may represent a pathologic diagnostic term to both benign and malignant mucinous neoplasms that produce abundant extracellular mucin. Therefore, poorly predictable clinical course and variable prognosis could be expected. A definitive diagnosis of PMP requires the presence of mucinous neoplastic epithelium and mucinous ascites, and may also include the diffuse mucinous implants [1]. In spite of more detailed understanding of PMP based on the clinical case series, there is still some debate about its clinical behavior, pathogenesis, and treatment strategy. We report our clinical experience concerning systemic chemotherapy (FOLFOX4 regimen) for one case of pseudomyxoma peritonei secondary to appendiceal mucinous adenocarcinoma and review the literature.

## Case presentation

**Figure 1:**
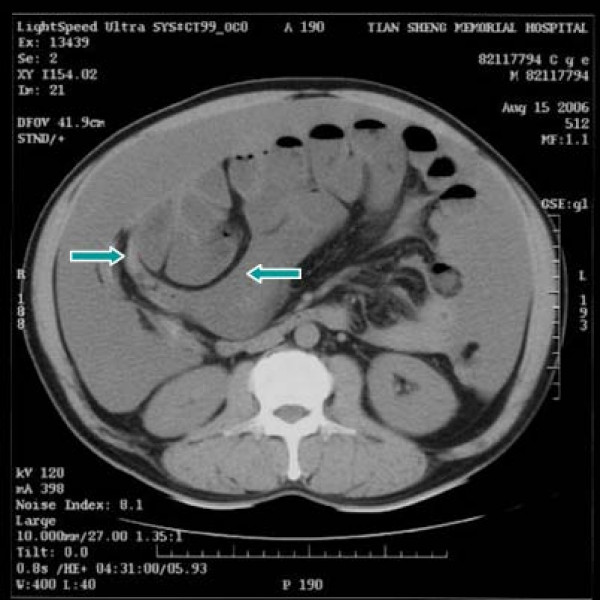
CT scan of abdomen showing pseudomyxoma peritonei with mucin septations (arrows).

**Figure 2:**
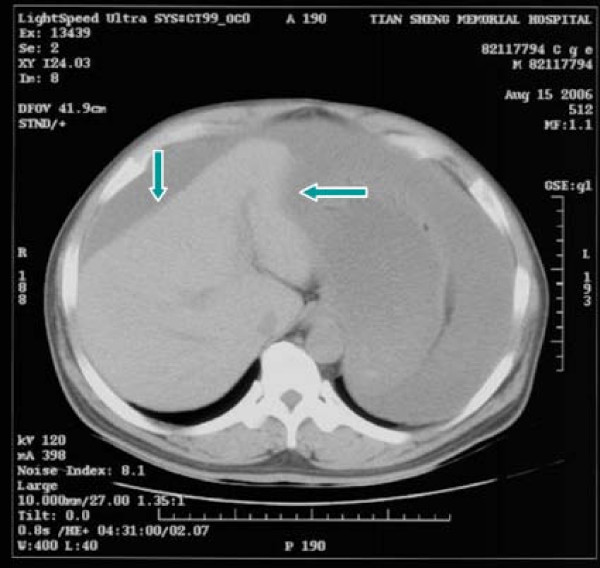
CT scan of abdomen showing pseudomyxoma peritonei with scalloping of hepatic margin (arrows).

**Figure 3:**
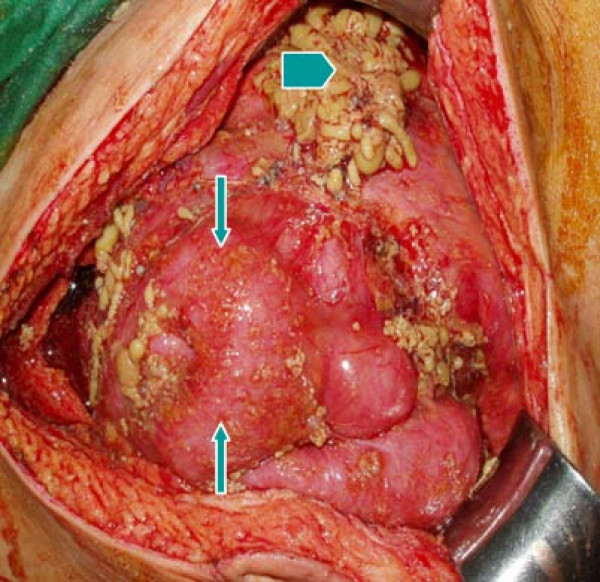
**Multiple peritoneal implants (arrows) over visceral peritoneum**. Mucinous ascites with yellowish-greenish materials (arrow head) in peritoneal cavity.

**Figure 4:**
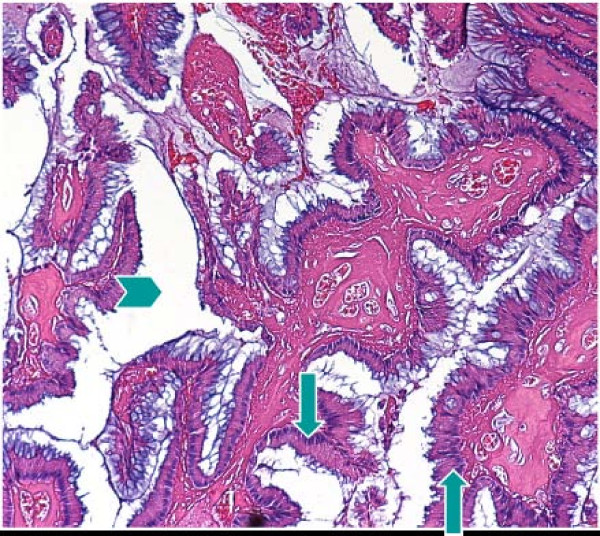
**The pathological findings of the resected appendix**. Mucinous adenocarcinoma (arrows) exhibiting abundant acellular mucin pooling (arrow head), with scarce well-differentiated mucin producing epithelium embedded in a fibrous matrix or as lining epithelium.

**Figure 5:**
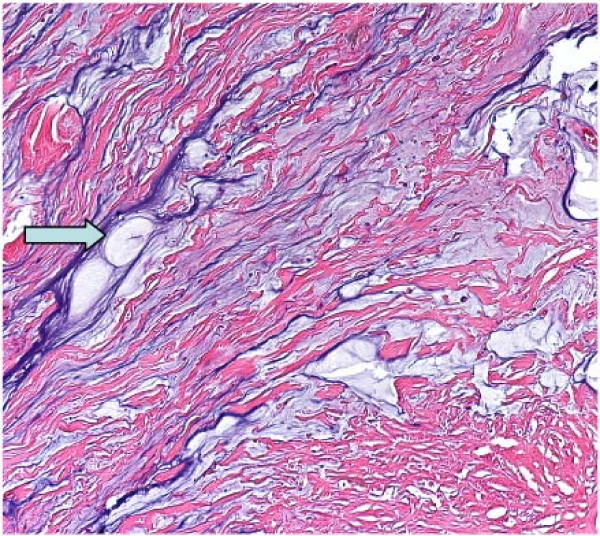
The pathological findings of the resected appendix extra-cellular mucinous materials (arrow) showing light blue in color distributed in fibrous stroma.

**Figure 6:**
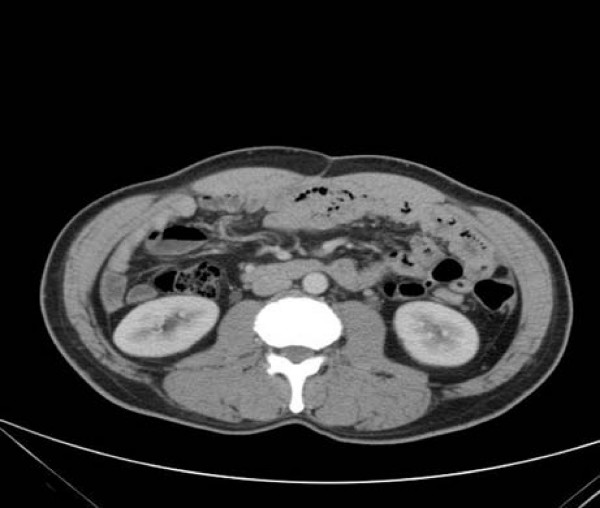
**CT scan of abdomen – postoperative 17 months, compared with Fig **1. No local recurrence or metastatic lesion is identified.

**Figure 7:**
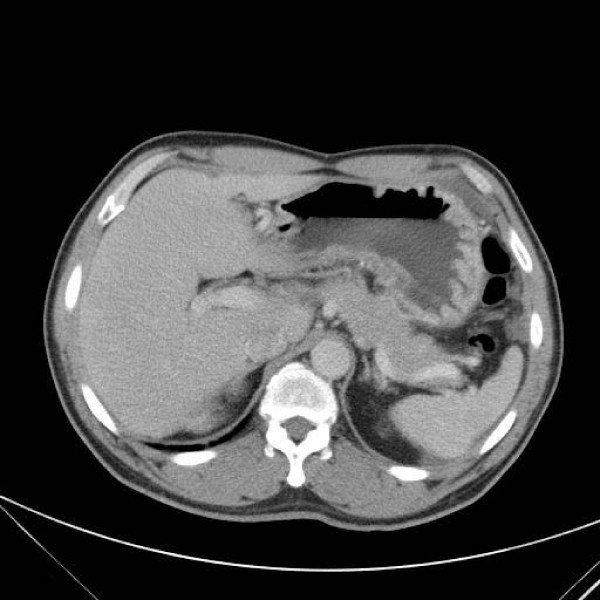
**CT scan of abdomen – postoperative 17 months, compared with Fig **2. No local recurrence or metastatic lesion is identified.

A 55-year-old male patient had suffered from acute onset of abdominal pain and abdominal distension for one day prior to his admission. He had previously been healthy without any specific underlying disease. Unfortunately, nausea and vomiting were noted twelve hours after his onset of abdominal pain. There was no fever, chills, or diarrhea. The characteristics of his abdominal pain included steady dull pain over the periumbilical and lower abdomen. On general physical examination, we found the patient presenting abdominal distension, hypoactive bowel sound, and diffuse tenderness over the whole abdomen, and localized muscle guarding over the right lower abdomen. Laboratory data showed predominant neutrophil (94%) without leukocytosis (white blood cell count 9160/μl). A large amount of ascites was identified by abdominal sonography and paracentesis was done. It showed the appearance of sticky mucinous ascites with the result of monocyte predominant in the ascites study. Abdominal computed tomography (CT) showed mucin septations (Fig. 1), thickening of the omentum and scalloping of hepatic and splenic margins (Fig. 2), which were compatible with the characteristics of the image of pseudomyxoma peritonei (PMP). He underwent laparotomy and much yellowish-greenish jelly-like material in the peritoneal cavity was noted (Fig. 3). Besides, one appendiceal tumor and multiple peritoneal implants disseminated from subphrenic space to the recto-vesicle pouch. Intra-operative frozen section of appendiceal tumor and one of the peritoneal implants confirmed mucinous adenocarcinoma of the appendix (Fig. 4 &5). The diagnosis of PMP was confirmed by the final histopathology. Instead of the aggressive peritonectomy procedures, we performed excision of the appendiceal tumor; local debridement and copious irrigation. After the operation, he received 10 cycles of systemic chemotherapy with FOLFOX4 regimen (oxaliplatin 85 mg/m^2 ^as a two-hour infusion on day 1, leucovorin 200 mg/m^2 ^as a two-hour infusion concurrently with oxaliplatin on day 1, followed by a bolus of 5-FU 400 mg/m^2 ^and continuous infusion of 5-FU 600 mg/m^2 ^over 22 hours), without specific morbidity [2]. Abdominal CT and colonoscopy at post-operative 17 months showed complete response without evidence of local recurrence or distal metastasis (Fig. 6 &7). Follow-up serum carcinoembryonic antigen (CEA) level also showed no progressive activity of his disease. He remains well currently, and receives follow-up regularly in our outpatient clinic.

## Discussion

Pseudomyxoma peritonei (PMP) is a rare clinical syndrome with an estimated incidence of approximately one per million per year and preferentially affects women (2–3 times more common than men) [3,4]. Since Werth [5] first described PMP produced by an ovarian neoplasm and Frankel [6] first reported on the association of PMP with an appendiceal mucocele, there have been many reports focusing on the pathogenesis, diagnosis, treatment and prognosis of PMP.

The primary origin of the mucinous peritoneal implants in PMP has remained controversial for a long time. Some reports indicated that ovarian tumor is more likely to be the primary neoplasm of PMP [3,7], however, others favor the appendiceal tumor as the answer [4,8,9]. Because immunohistochemical stains and molecular genetic studies both show the evidence of these tumors being secondary to appendiceal neoplasms [10-12], most people agree that the primary tumor of PMP is predominately a mucinous epithelial neoplasm of the appendix. Other possible primary sites being reported include: colorectum, gallbladder, pancreas, urachus, urinary bladder, breast and lung, but these are uncommon [13,14]. PMP is characterized by an abundant amount of mucinous ascites produced by adenomucinous tumor cells in implants on peritoneal surfaces. These implants are the final stage of a distribution process following the rupture of the mucinous neoplasm. The key associated finding is the presence of epithelium outside the appendix in association with the mucin and the peritoneal implants [1].

Ronnett et al [15] first described a widely accepted and useful definition of PMP. They classified PMP into three pathological subtypes with different pathological characteristics and different prognosis: disseminated peritoneal adenomucinosis (DPAM), peritoneal mucinous carcinomatosis (PMCA), and an intermediate subtype (PMCA-I). Histopathologically, DPAM is characterized by an abundance of mucus with focally adenomucinous epithelium without atypia or mitotic activity. In contrast to this, PMCA is characterized by peritoneal tumor composed of more abundant mucinous tumor cells with the architecture and cytological features of carcinoma. Finally, the intermediate subtype PMCA-I is characterized by an abundance of DPAM lesions, but with focal areas with PMCA lesions [15,16].

Besides the histopathological difference, their clinical behaviors are also quite different. DPAM remains potentially non-invasive and stays localized to the abdomen without metastatic behavior. In contrast to this, PMCA behaves with invasive and metastatic potential, as is characteristic of mucinous adenocarcinoma. Patients with PCMA are associated with the possibilities of liver, lung and lymph node metastatic disease [17].

As symptoms remain non-specific, PMP presents a great diagnostic challenge to clinicians. A precise diagnosis is difficult due to the lack of specific symptoms in the early stages of the disease. The most important symptom is a gradually increasing abdominal girth. Patients may present a typical "jelly belly" appearance [18]. Sometimes patients present symptoms mimicking appendicitis with intra-operative identification of a perforated appendiceal mucocele. In other cases, they present an inguinal herniated sac or an ovarian mass [18]. For 30% of female patients, the first symptom is an ovarian mass [16].

Routine laboratory studies are seldom helpful in making this diagnosis. Ultrasound is useful for initial establishment of the diagnosis. Echo-guided paracentesis may reveal mucinous ascites. Abdominal computed tomography (CT) scan may demonstrate the characteristics of mucinous ascites by analyzing the density properties (Hounsfield Units [H.U.]), as it is significantly higher (5–20 H.U.) than normal ascites (0 H.U.) [16]. CT may also show the characteristic of "scalloping effect" on the surface of the visceral organs resulting from compression by the viscous mucinous secretions [1]. In the majority of cases, however, PMP is often an unexpected finding of laparoscopy or exploratory laparotomy [19].

When mucinous tumors on the peritoneal surface or mucinous ascites are visualized on CT or during abdominal surgery, treatment of PMP should be performed since untreated PMP patients will eventually suffer from intestinal obstruction and mortality [20]. In spite of the controversial standard treatment strategies for PMP, the current mainstay of the treatment remains surgical resection of the lesions. Alternative non-surgical treatment, such as: peritoneal washing with 5% dextrose and systemic chemotherapy, have been reported; however, their roles in PMP are still uncertain because of the limited case number and short follow-up time [21,22].

Repeated cytoreductive surgical de-bulking procedures as treatment for PMP have been described in earlier literatures. Since the 1990s, a new combined treatment approach was introduced by Sugarbaker et al [23]. They defined it as peritonectomy procedures in combination with intra-operative hyperthermic intra-peritoneal chemotherapy (HIPEC). Now this new combination treatment is increasingly performed as treatment for PMP patients, with promising results [24,25]. The available evidence suggests that cytoreductive surgery with perioperative intraperitoneal chemotherapy should replace serial de-bulking as the standard of care for patients with peritoneal spread of appendiceal epithelial neoplasms [20]. PMCA behaves like peritoneal carcinomatosis from the original colorectal adenocarcinoma; however, its poor prognosis in comparison with DPAM after the similar management by cytoreductive surgery and HIPEC was still demonstrated in the two studies conducted by Sugarbaker et al [26]. and Smeenk et al [27]. Moreover, Verwaal et al, also showed a similar result in their randomized study, that patients with peritoneal carcinomatosis of colorectal cancer (CRC) origin combined with cancer implant involvement of six or more regions of the abdominal cavity, got little survival benefit after the cytoreductive surgical procedures and intra-operative HIPEC [28].

Herein, further clinical trials with investigations of different treatment strategies for patients with PMCA, are still needed. This contributes to the application of systemic chemotherapy for the patient in our case report since the new therapeutic agent Oxaliplatin and its combination with 5-fluorouracil/leucovorin (FOLFOX4 regimen) has been used widely as first-line treatment in patients with advanced CRC, and the promising results in these patients have been demonstrated in several randomized studies [29,30].

The effect of systemic chemotherapy in PMP seems questionable. Jones et al [22] reported their experience in the treatment of pseudomyxoma peritonei of ovarian origin with cisplatinum, doxorubicin, and cyclophosphamide, with excellent responses. On the other hand, Smeenk et al [31] reported the poor response of six patients (3 patients with DPAM, another 3 patients with PMCA-I, and all 6 patients with lesions diffusely spread throughout the abdomen) after 5-FU based systemic chemotherapy, and subsequent poor prognosis was noted in the study. Regarding the benefit of new therapeutic agents (including Capecitabine, Oxaliplatin, Irinotecan and Bevacizumab) and modern schedules for patients with metastatic CRC, clinical experience with the use of these agents for PMP are still absent, and it is questionable whether they will do any better in this situation, especially for patients with PMCA. Due to the limited experience and indeterminate effects of systemic chemotherapy in PMP, some studies still suggest that systemic therapy should be reserved for a palliative setting in patients with recurrent or progressive disease [20,32].

Because of the high grade mucinous adenocarcinoma of the appendix with disseminated peritoneal lesions both confirmed by histopathology, instead of the treatment strategies (including aggressive peritonectomy procedures), we used oxaliplatin/5-FU/leucovorin combination systemic chemotherapy (FOLFOX4 regimen) in our patient after less invasive surgery, as the treatment for the metastatic colorectal cancer. After 10 cycles of FOLFOX4 chemotherapy, excellent response was achieved. Colonoscopy and abdominal CT scan 17 months after the operation both showed no evidence of development of local recurrent or metastatic lesions. The patient had an uneventful recovery up to now without any disease-related morbidity. We believe that our experience is one of the few reports about effective treatment of PMP with systemic chemotherapy. More clinical experience and further studies are still needed for determination of the benefit of systemic chemotherapy for these patients.

## Conclusion

We report our clinical experience regarding the use of systemic chemotherapy (FOLFOX4 regimen) for one case of pseudomyxoma peritonei secondary to mucinous adenocarcinoma of the appendix. This case report emphasizes the possible new role of systemic chemotherapy in the treatment of patients with this rare clinical syndrome.

## Consent

Written informed consent was taken from the patient for publication of this case report.

## Competing interests

The authors declare that they have no competing interests.

## Authors' contributions

CFC performed the initial surgery, conceptualized the case report, gathered the data, reviewed the literature and drafted the manuscript. CJH performed the initial surgery, took responsibility for the patient's postoperative care and revised the manuscript. WYK assessed the histological specimens and prepared the histological slides. JSH reviewed the clinical data and helped to draft and revise the manuscript. All authors read and approved the final manuscript.
